# 4-Phenyl­sulfon­yl-2-(*p*-tolyl­sulfon­yl)-1*H*,8*H*-pyrrolo­[2,3-*b*]indole

**DOI:** 10.1107/S1600536810039425

**Published:** 2010-10-09

**Authors:** Jeanese C. Badenock, Jason A. Jordan, Erin T. Pelkey, Gordon W. Gribble, Jerry P. Jasinski

**Affiliations:** a Department of Biological and Chemical Sciences, University of the West Indies, Cave Hill, Barbados; b Department of Chemistry, Dartmouth College, Hanover, NH 03755-3564, USA; c Department of Chemistry, Keene State College, 229 Main Street, Keene, NH 03435-2001, USA

## Abstract

The title compound, C_23_H_18_N_2_O_4_S_2_, contains a pyrrolo group fused onto the plane of an indole ring with phenyl­sulfonyl and *p*-toluene­sulfonyl groups bonded to the indole and pyrrolo rings. The angles between the mean planes of the pyrrolo-indole ring and the phenyl­sulfonyl and *p*-toluene­sulfonyl rings are 73.7 (6) and 80.6 (0)°, respectively. The dihedral angle between the mean planes of the two benzene rings is 78.7 (4)°. In the crystal, both classical N—H⋯O and non-classical C—H⋯O inter­molecular hydrogen-bonding inter­actions are observed, as well as weak π–π inter­actions [centroid–centroid distances = 3.6258 (8) and 3.9298 (8) Å], which contribute to the stability of the packing.

## Related literature

We have been inter­ested in the synthesis of fused indole heterocycles (Gribble *et al.*, 2005 ▶) for the construction of more elaborate mol­ecules, such as the potent anti­biotics pyrroindomycins A and B (Abbanat *et al.*, 1999 ▶; Ding *et al.*, 1994 ▶) Both pyrrolo­[2,3-*b*]indoles and pyrrolo­[3,4-*b*]indoles can be synthesized in one step *via* the Barton–Zard pyrrole synthesis (Barton & Zard, 1985 ▶; Barton *et al.*, 1990 ▶) from 3-nitro­indoles, depending on the *N*-indole protecting group [Pelkey *et al.*, 1996 ▶; Pelkey & Gribble, 1997 ▶, 1999 ▶, 2006 ▶). For recent examples of the Barton–Zard pyrrole synthesis, see: Bobal & Lightner (2001 ▶); Woydziak *et al.* (2005 ▶); Larionov & deMeijere (2005 ▶); Coffin *et al.* (2006 ▶); Okujima *et al.* (2006 ▶); Ono (2008 ▶). For related structures, see: Jackson *et al.* (1975 ▶); Moody & Ward (1984*a* ▶,*b* ▶); Yamane *et al.* (1986 ▶); Yin *et al.* (2010 ▶); Tsuji *et al.* (2002 ▶); Somei *et al.* (1997 ▶); Kawasaki *et al.* (2005 ▶); Jasinski *et al.* (2010 ▶). For MOPAC theoretical calculations, see: Schmidt & Polik (2007 ▶). For standard bond lengths, see: Allen *et al.* (1987 ▶)
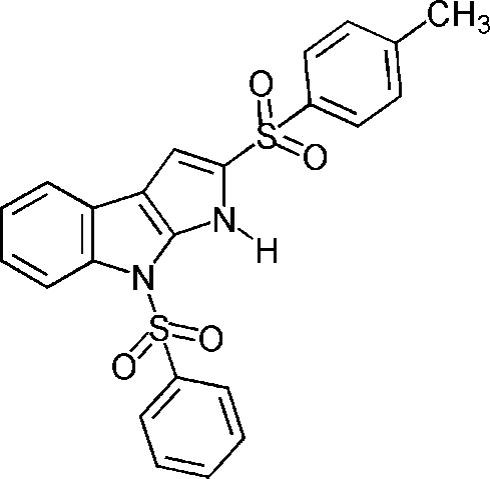


## Experimental

### 

#### Crystal data

C_23_H_18_N_2_O_4_S_2_
*M* *_r_* = 450.51
Triclinic, 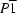

*a* = 8.1547 (3) Å
*b* = 11.0471 (5) Å
*c* = 11.7185 (4) Å
α = 73.834 (4)°
β = 87.131 (3)°
γ = 79.277 (4)°
*V* = 996.22 (7) Å^3^
*Z* = 2
Mo *K*α radiation
μ = 0.30 mm^−1^
*T* = 123 K
0.41 × 0.36 × 0.29 mm

#### Data collection

Oxford Diffraction Xcalibur diffractometer with Ruby (Gemini) detector
Absorption correction: multi-scan (*CrysAlis RED*; Oxford Diffraction, 2007 ▶) *T*_min_ = 0.981, *T*_max_ = 1.000
12580 measured reflections
6592 independent reflections
5331 reflections with *I* > 2σ(*I*)
*R*_int_ = 0.020

#### Refinement

*R*[*F* ^2^ > 2σ(*F* ^2^)] = 0.036
*wR*(*F* ^2^) = 0.103
*S* = 1.09
6592 reflections
281 parameters
H-atom parameters constrained
Δρ_max_ = 0.52 e Å^−3^
Δρ_min_ = −0.33 e Å^−3^

### 

Data collection: *CrysAlis PRO* (Oxford Diffraction, 2007 ▶); cell refinement: *CrysAlis PRO*; data reduction: *CrysAlis RED* (Oxford Diffraction, 2007 ▶); program(s) used to solve structure: *SHELXS97* (Sheldrick, 2008 ▶); program(s) used to refine structure: *SHELXL97* (Sheldrick, 2008 ▶); molecular graphics: *SHELXTL* (Sheldrick, 2008 ▶); software used to prepare material for publication: *SHELXTL*.

## Supplementary Material

Crystal structure: contains datablocks global, I. DOI: 10.1107/S1600536810039425/fl2319sup1.cif

Structure factors: contains datablocks I. DOI: 10.1107/S1600536810039425/fl2319Isup2.hkl

Additional supplementary materials:  crystallographic information; 3D view; checkCIF report

## Figures and Tables

**Table 1:** Hydrogen-bond geometry (Å, °)

*D*—H⋯*A*	*D*—H	H⋯*A*	*D*⋯*A*	*D*—H⋯*A*
N2—H2*B*⋯O3^i^	0.88	2.06	2.9244 (14)	167
C13—H13*A*⋯O2^ii^	0.95	2.53	3.2125 (15)	129
C22—H22*A*⋯O3^i^	0.95	2.45	3.3786 (15)	165

Symmetry codes: (i) 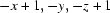; (ii) 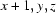.

**Fig. 1.**
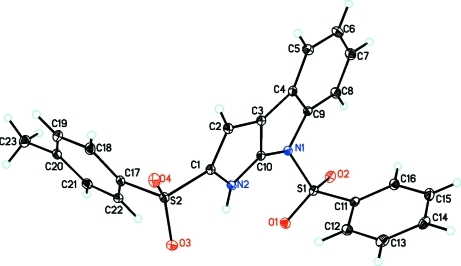
Molecular structure of the title compound showing the atom labeling scheme and 50% probability displacement ellipsoids.

**Fig. 2.**
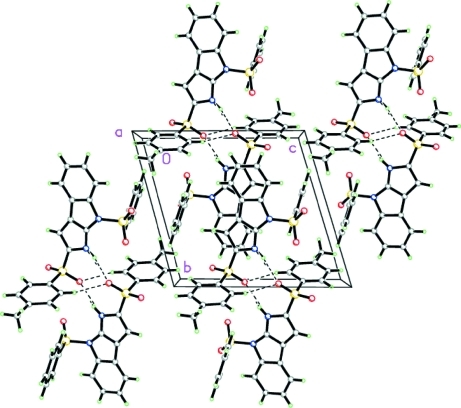
Packing diagram of the title compound viewed down the a axis. Dashed lines indicate classical N—H···O and non-classical C—H···O hydrogen bonds with a bifurcated O3 acceptor atom.

## supplementary crystallographic information

### Comment

In view of our continued interest in the synthesis of fused indole heterocycles (Gribble *et al.*, 2005) for the construction of more elaborate molecules, such as the potent antibiotics pyrroindomycins A and B (Abbanat *et al.*, 1999; Ding *et al.*, 1994), we have sought to unequivocally confirm the assigned structure of the product formed in the reaction of 3-nitro-1-(phenylsulfonyl)indole with isocyanide. Our previous studies have shown that both pyrrolo[2,3-*b*]indoles and pyrrolo[3,4-*b*]indoles can be synthesized in one step *via* this Barton-Zard pyrrole synthesis (Barton & Zard, 1985; Barton *et al.*, 1990) from 3-nitroindoles depending on the *N*-indole protecting group (Pelkey *et al.*, 1996; Pelkey & Gribble, 1997, 1999, 2006). Indeed, whereas our proposed fragmentation-rearrangement sequence, to afford the pyrrolo[2,3-*b*]indole ring system (Pelkey *et al.*, 1996), only occurs with the phenylsulfonyl protecting group, the same reaction with *N*-benzyl, N-2-pyridyl, and *N*-ethoxycarbonyl protecting groups generates the corresponding pyrrolo[3,4-*b*]indole ring system. We differentiated these two isomers both by comparison of the NMR coupling constants and through the independent synthesis of the corresponding pyrrolo[2,3-*b*]indole. (Moody & Ward, 1984*a*, 1984*b*). To confirm this structural assignment we now report the crystal structure of the title compound, the product of the reaction of 3-nitro-1-(phenylsulfonyl)indole with tosylmethyl isocyanide, and the first crystal structure of this fused indole ring system.

The title compound contains a pyrrolo group fused onto the plane of an indole ring with phenylsulfonyl and *p*-toluenesulfonyl groups bonded to the indol and pyrrolo rings. The angles between the mean planes of the pyrrolo-indole ring and the phenylsulfonyl and *p*-toluenesulfonyl rings are 73.7 (6)° and 80.6 (0)°, respectively. The dihedral angle between the mean planes of the two benzene rings is 78.7 (4)°. The sum of the angles around the indole N atom is 345.2 (4)° indicating slightly distorted *sp*^2^ hybridization. The C3═C10 indole bond length is 1.3760 (17)Å similar to that observed in 3-acetyl-2-ethyl-1-(phenylsulfonyl)indole (Jasinski *et al.*, 2010). The remainder of the bonds are in normal ranges (Allen *et al.*, 1987). Both classical (N—H···O) and non-classical (C—H···O) hydrogen bonding interactions are observed (Table 1, Fig. 2) as well as weak π—π interactions [*Cg*1···*Cg*3^i^ = 3.6258 (8) Å; *Cg*2···*Cg*3^i^ = 3.9298 (8) Å; ^i^ = -*x*,1 - *y*,1 - *z*; where *Cg*1 = N1/C9/C4/C3/C10; *Cg*2 = N2/C1/C2/C3/C10; *Cg*3 = C4—C9].

Following geometry optimization MOPAC (Schmidt & Polik, 2007) theoretical calculations at the AM1 level, the angles between the mean planes of the pyrrolo-indole ring and the phenylsulfonyl and *p*-toluenesulfonyl rings become 73.7 (6)° and 80.6 (0)°, respectively, and the dihedral angle between the mean planes of the two benzene rings becomes 88.6 (2)°. These observations support the influence of the hydrogen bonds and π—π interactions as contributing to the stability of crystal packing.

### Experimental

This compound was prepared according to the procedure of Pelkey & Gribble (2006). To a stirred solution of 3-nitro-1-(phenylsulfonyl)indole (0.50 g, 1.67 mmol, 1 eq.) in dry THF (30 ml) was added a solution of tosylmethyl isocyanide (0.39 g, 1.99 mmol, 1.20 eq.) dissolved in dry THF (15 ml) followed by the addition of DBU (0.6 ml, 4.01 mmol, 2.4 eq.). The solution was allowed to stir for 24 h at room temperature. Removal of the solvent in vacuo gave an orange oil that was purified *via* flash column chromatography (3:1 hexanes–ethyl acetate) to afford the pyrroloindole (0.46 g, 62%) as a yellow solid. Crystals suitable for the X-ray study were grown from a 1:1 mixture of CH_2_Cl_2_ and ether [m.p. 484–487 K; literature value 509–511 K].

### Refinement

All the H atoms were discernible in the difference electron density map, however, they were situated into idealized positions. The parameters of all the H atoms have been constrained within the riding atom approximation. C—H bond lengths were constrained to 0.95 or 0.98 Å for aryl or methyl H atoms, and 0.88 for N—H atoms, *U*_iso_(H) = 1.17–1.22*U*_eq_(C_aryl_); *U*_iso_(H) = 1.51*U*_eq_(C_methyl_) or *U*_iso_(H) = 1.16*U*_eq_(N).

### Crystal data

**Table:**

C_23_H_18_N_2_O_4_S_2_	*Z* = 2
*M**_r_* = 450.51	*F*(000) = 468
Triclinic, *P*1	*D*_x_ = 1.502 Mg m^−^^3^
Hall symbol: -P 1	Mo *K*α radiation, λ = 0.71073 Å
*a* = 8.1547 (3) Å	Cell parameters from 7126 reflections
*b* = 11.0471 (5) Å	θ = 5.0–32.7°
*c* = 11.7185 (4) Å	µ = 0.30 mm^−^^1^
α = 73.834 (4)°	*T* = 123 K
β = 87.131 (3)°	Prism, colorless
γ = 79.277 (4)°	0.41 × 0.36 × 0.29 mm
*V* = 996.22 (7) Å^3^	

### Data collection

**Table:**

Oxford Diffraction Xcalibur diffractometer with Ruby (Gemini) detector	6592 independent reflections
Radiation source: Enhance (Cu) X-ray Source	5331 reflections with *I* > 2σ(*I*)
graphite	*R*_int_ = 0.020
Detector resolution: 10.5081 pixels mm^-1^	θ_max_ = 32.8°, θ_min_ = 5.1°
ω scans	*h* = −11→11
Absorption correction: multi-scan (*CrysAlis RED*; Oxford Diffraction, 2007)	*k* = −16→16
*T*_min_ = 0.981, *T*_max_ = 1.000	*l* = −17→13
12580 measured reflections	

### Refinement

**Table:**

Refinement on *F*^2^	Primary atom site location: structure-invariant direct methods
Least-squares matrix: full	Secondary atom site location: difference Fourier map
*R*[*F*^2^ > 2σ(*F*^2^)] = 0.036	Hydrogen site location: inferred from neighbouring sites
*wR*(*F*^2^) = 0.103	H-atom parameters constrained
*S* = 1.09	*w* = 1/[σ^2^(*F*_o_^2^) + (0.0572*P*)^2^ + 0.0394*P*] where *P* = (*F*_o_^2^ + 2*F*_c_^2^)/3
6592 reflections	(Δ/σ)_max_ < 0.001
281 parameters	Δρ_max_ = 0.52 e Å^−^^3^
0 restraints	Δρ_min_ = −0.33 e Å^−^^3^

### Special details

**Table:**

Geometry. All e.s.d.'s (except the e.s.d. in the dihedral angle between two l.s. planes) are estimated using the full covariance matrix. The cell e.s.d.'s are taken into account individually in the estimation of e.s.d.'s in distances, angles and torsion angles; correlations between e.s.d.'s in cell parameters are only used when they are defined by crystal symmetry. An approximate (isotropic) treatment of cell e.s.d.'s is used for estimating e.s.d.'s involving l.s. planes.
Refinement. Refinement of *F*^2^ against ALL reflections. The weighted *R*-factor *wR* and goodness of fit *S* are based on *F*^2^, conventional *R*-factors *R* are based on *F*, with *F* set to zero for negative *F*^2^. The threshold expression of *F*^2^ > σ(*F*^2^) is used only for calculating *R*-factors(gt) *etc*. and is not relevant to the choice of reflections for refinement. *R*-factors based on *F*^2^ are statistically about twice as large as those based on *F*, and *R*- factors based on ALL data will be even larger.

### Fractional atomic coordinates and isotropic or equivalent isotropic displacement parameters (Å^2^)

**Table:**

	*x*	*y*	*z*	*U*_iso_*/*U*_eq_	
S1	0.24124 (3)	0.40940 (3)	0.21069 (3)	0.01455 (7)	
S2	0.53714 (4)	0.07067 (3)	0.67939 (3)	0.01540 (7)	
O1	0.28990 (11)	0.27445 (8)	0.22946 (8)	0.01961 (18)	
O2	0.10331 (10)	0.48046 (9)	0.13513 (8)	0.01992 (19)	
O3	0.61740 (11)	−0.00088 (8)	0.59944 (8)	0.02005 (19)	
O4	0.63277 (11)	0.09255 (9)	0.76857 (8)	0.02102 (19)	
N1	0.19171 (12)	0.42667 (9)	0.34605 (9)	0.01529 (19)	
N2	0.36818 (12)	0.22963 (9)	0.48254 (9)	0.01541 (19)	
H2B	0.3722	0.1694	0.4462	0.018*	
C1	0.43829 (15)	0.21752 (11)	0.59259 (10)	0.0155 (2)	
C2	0.40399 (15)	0.33305 (11)	0.62081 (11)	0.0169 (2)	
H2A	0.4367	0.3501	0.6906	0.020*	
C3	0.30982 (15)	0.42085 (11)	0.52414 (11)	0.0160 (2)	
C4	0.21746 (15)	0.55003 (11)	0.47483 (11)	0.0161 (2)	
C5	0.18616 (17)	0.66151 (12)	0.51200 (12)	0.0218 (3)	
H5A	0.2306	0.6628	0.5850	0.026*	
C6	0.08910 (17)	0.77060 (12)	0.44084 (12)	0.0242 (3)	
H6A	0.0680	0.8471	0.4653	0.029*	
C7	0.02218 (16)	0.76964 (12)	0.33428 (12)	0.0232 (3)	
H7A	−0.0436	0.8457	0.2871	0.028*	
C8	0.04966 (15)	0.65990 (12)	0.29554 (12)	0.0200 (2)	
H8A	0.0027	0.6588	0.2233	0.024*	
C9	0.14851 (14)	0.55177 (11)	0.36653 (11)	0.0159 (2)	
C10	0.29295 (14)	0.35350 (11)	0.44405 (10)	0.0146 (2)	
C11	0.41636 (14)	0.48258 (11)	0.16669 (10)	0.0142 (2)	
C12	0.57366 (15)	0.40983 (12)	0.20143 (11)	0.0186 (2)	
H12A	0.5850	0.3233	0.2480	0.022*	
C13	0.71326 (15)	0.46588 (13)	0.16684 (12)	0.0214 (3)	
H13A	0.8213	0.4177	0.1901	0.026*	
C14	0.69552 (16)	0.59186 (13)	0.09850 (11)	0.0210 (3)	
H14A	0.7918	0.6295	0.0746	0.025*	
C15	0.53830 (17)	0.66374 (13)	0.06448 (12)	0.0214 (3)	
H15A	0.5276	0.7502	0.0176	0.026*	
C16	0.39661 (15)	0.60964 (12)	0.09882 (11)	0.0184 (2)	
H16A	0.2886	0.6584	0.0764	0.022*	
C17	0.37346 (15)	−0.00606 (11)	0.75228 (10)	0.0155 (2)	
C18	0.34028 (16)	−0.00884 (12)	0.87037 (11)	0.0188 (2)	
H18A	0.4056	0.0289	0.9110	0.023*	
C19	0.21030 (16)	−0.06741 (12)	0.92891 (11)	0.0197 (2)	
H19A	0.1885	−0.0706	1.0100	0.024*	
C20	0.11216 (15)	−0.12121 (11)	0.86952 (11)	0.0175 (2)	
C21	0.14575 (16)	−0.11501 (12)	0.74970 (11)	0.0188 (2)	
H21A	0.0777	−0.1496	0.7080	0.023*	
C22	0.27672 (15)	−0.05922 (11)	0.69078 (11)	0.0178 (2)	
H22A	0.3001	−0.0573	0.6101	0.021*	
C23	−0.02767 (17)	−0.18520 (13)	0.93246 (13)	0.0244 (3)	
H23A	−0.0415	−0.1727	1.0122	0.037*	
H23B	−0.0010	−0.2771	0.9388	0.037*	
H23C	−0.1315	−0.1475	0.8873	0.037*	

### Atomic displacement parameters (Å^2^)

**Table:**

	*U*^11^	*U*^22^	*U*^33^	*U*^12^	*U*^13^	*U*^23^
S1	0.01309 (13)	0.01663 (14)	0.01399 (14)	−0.00350 (10)	−0.00090 (10)	−0.00358 (10)
S2	0.01639 (14)	0.01312 (13)	0.01499 (14)	−0.00131 (10)	0.00004 (10)	−0.00193 (10)
O1	0.0238 (4)	0.0173 (4)	0.0193 (4)	−0.0061 (3)	−0.0006 (3)	−0.0059 (3)
O2	0.0137 (4)	0.0273 (5)	0.0173 (4)	−0.0036 (3)	−0.0034 (3)	−0.0031 (4)
O3	0.0205 (4)	0.0163 (4)	0.0219 (5)	−0.0005 (3)	0.0043 (3)	−0.0056 (3)
O4	0.0204 (4)	0.0218 (4)	0.0201 (5)	−0.0053 (4)	−0.0037 (3)	−0.0028 (4)
N1	0.0165 (5)	0.0145 (4)	0.0133 (5)	−0.0013 (4)	0.0001 (4)	−0.0022 (4)
N2	0.0189 (5)	0.0120 (4)	0.0147 (5)	−0.0012 (4)	−0.0003 (4)	−0.0036 (4)
C1	0.0183 (5)	0.0139 (5)	0.0127 (5)	−0.0018 (4)	0.0003 (4)	−0.0017 (4)
C2	0.0211 (6)	0.0146 (5)	0.0143 (5)	−0.0028 (4)	0.0004 (4)	−0.0030 (4)
C3	0.0187 (5)	0.0139 (5)	0.0145 (5)	−0.0024 (4)	0.0018 (4)	−0.0033 (4)
C4	0.0167 (5)	0.0141 (5)	0.0156 (5)	−0.0025 (4)	0.0029 (4)	−0.0018 (4)
C5	0.0275 (7)	0.0167 (5)	0.0203 (6)	−0.0016 (5)	0.0019 (5)	−0.0054 (5)
C6	0.0270 (7)	0.0148 (6)	0.0287 (7)	0.0000 (5)	0.0040 (5)	−0.0059 (5)
C7	0.0203 (6)	0.0169 (6)	0.0271 (7)	0.0024 (5)	0.0020 (5)	−0.0014 (5)
C8	0.0175 (6)	0.0186 (6)	0.0201 (6)	0.0007 (5)	−0.0008 (4)	−0.0014 (5)
C9	0.0149 (5)	0.0143 (5)	0.0173 (6)	−0.0019 (4)	0.0040 (4)	−0.0033 (4)
C10	0.0153 (5)	0.0134 (5)	0.0139 (5)	−0.0024 (4)	0.0013 (4)	−0.0020 (4)
C11	0.0120 (5)	0.0178 (5)	0.0134 (5)	−0.0037 (4)	0.0006 (4)	−0.0046 (4)
C12	0.0160 (5)	0.0184 (5)	0.0191 (6)	−0.0007 (4)	−0.0011 (4)	−0.0029 (5)
C13	0.0129 (5)	0.0288 (7)	0.0222 (6)	−0.0022 (5)	−0.0004 (4)	−0.0073 (5)
C14	0.0178 (6)	0.0301 (7)	0.0187 (6)	−0.0112 (5)	0.0043 (4)	−0.0085 (5)
C15	0.0253 (6)	0.0202 (6)	0.0177 (6)	−0.0073 (5)	0.0011 (5)	−0.0019 (5)
C16	0.0177 (5)	0.0188 (5)	0.0165 (6)	−0.0016 (4)	−0.0013 (4)	−0.0019 (4)
C17	0.0171 (5)	0.0114 (5)	0.0153 (5)	−0.0007 (4)	0.0000 (4)	−0.0006 (4)
C18	0.0227 (6)	0.0174 (5)	0.0153 (6)	−0.0035 (5)	−0.0030 (4)	−0.0024 (4)
C19	0.0235 (6)	0.0200 (6)	0.0141 (6)	−0.0035 (5)	0.0010 (4)	−0.0026 (4)
C20	0.0190 (6)	0.0128 (5)	0.0184 (6)	−0.0013 (4)	0.0013 (4)	−0.0018 (4)
C21	0.0226 (6)	0.0151 (5)	0.0197 (6)	−0.0041 (4)	−0.0003 (5)	−0.0057 (4)
C22	0.0224 (6)	0.0148 (5)	0.0153 (6)	−0.0012 (4)	0.0018 (4)	−0.0044 (4)
C23	0.0258 (7)	0.0244 (6)	0.0241 (7)	−0.0098 (5)	0.0058 (5)	−0.0062 (5)

### Geometric parameters (Å, °)

**Table:**

S1—O1	1.4263 (9)	C8—H8A	0.9500
S1—O2	1.4316 (9)	C11—C16	1.3914 (16)
S1—N1	1.6707 (10)	C11—C12	1.3941 (16)
S1—C11	1.7557 (12)	C12—C13	1.3856 (18)
S2—O4	1.4322 (9)	C12—H12A	0.9500
S2—O3	1.4455 (9)	C13—C14	1.3839 (19)
S2—C1	1.7300 (12)	C13—H13A	0.9500
S2—C17	1.7701 (12)	C14—C15	1.3889 (18)
N1—C10	1.4084 (15)	C14—H14A	0.9500
N1—C9	1.4446 (15)	C15—C16	1.3895 (18)
N2—C10	1.3506 (14)	C15—H15A	0.9500
N2—C1	1.3990 (15)	C16—H16A	0.9500
N2—H2B	0.8800	C17—C18	1.3896 (17)
C1—C2	1.3818 (17)	C17—C22	1.3970 (17)
C2—C3	1.4184 (17)	C18—C19	1.3958 (17)
C2—H2A	0.9500	C18—H18A	0.9500
C3—C10	1.3760 (17)	C19—C20	1.3932 (18)
C3—C4	1.4567 (16)	C19—H19A	0.9500
C4—C5	1.3937 (18)	C20—C21	1.4022 (17)
C4—C9	1.4075 (17)	C20—C23	1.5065 (17)
C5—C6	1.3877 (18)	C21—C22	1.3897 (17)
C5—H5A	0.9500	C21—H21A	0.9500
C6—C7	1.391 (2)	C22—H22A	0.9500
C6—H6A	0.9500	C23—H23A	0.9800
C7—C8	1.3861 (19)	C23—H23B	0.9800
C7—H7A	0.9500	C23—H23C	0.9800
C8—C9	1.3871 (16)		
			
O1—S1—O2	121.13 (6)	N2—C10—C3	111.89 (11)
O1—S1—N1	104.32 (5)	N2—C10—N1	134.91 (11)
O2—S1—N1	106.30 (5)	C3—C10—N1	112.99 (10)
O1—S1—C11	109.10 (5)	C16—C11—C12	121.61 (11)
O2—S1—C11	109.09 (5)	C16—C11—S1	120.24 (9)
N1—S1—C11	105.77 (5)	C12—C11—S1	118.15 (9)
O4—S2—O3	120.17 (6)	C13—C12—C11	118.89 (11)
O4—S2—C1	108.32 (6)	C13—C12—H12A	120.6
O3—S2—C1	107.11 (6)	C11—C12—H12A	120.6
O4—S2—C17	107.49 (6)	C14—C13—C12	120.11 (12)
O3—S2—C17	107.91 (6)	C14—C13—H13A	119.9
C1—S2—C17	104.84 (6)	C12—C13—H13A	119.9
C10—N1—C9	103.92 (10)	C13—C14—C15	120.63 (12)
C10—N1—S1	119.83 (8)	C13—C14—H14A	119.7
C9—N1—S1	121.49 (8)	C15—C14—H14A	119.7
C10—N2—C1	105.31 (10)	C14—C15—C16	120.20 (12)
C10—N2—H2B	127.3	C14—C15—H15A	119.9
C1—N2—H2B	127.3	C16—C15—H15A	119.9
C2—C1—N2	110.47 (10)	C15—C16—C11	118.56 (11)
C2—C1—S2	128.02 (10)	C15—C16—H16A	120.7
N2—C1—S2	121.31 (9)	C11—C16—H16A	120.7
C1—C2—C3	105.85 (11)	C18—C17—C22	120.89 (11)
C1—C2—H2A	127.1	C18—C17—S2	118.71 (10)
C3—C2—H2A	127.1	C22—C17—S2	120.37 (9)
C10—C3—C2	106.48 (10)	C17—C18—C19	119.56 (12)
C10—C3—C4	106.28 (10)	C17—C18—H18A	120.2
C2—C3—C4	147.16 (12)	C19—C18—H18A	120.2
C5—C4—C9	118.78 (11)	C20—C19—C18	120.61 (12)
C5—C4—C3	134.52 (12)	C20—C19—H19A	119.7
C9—C4—C3	106.70 (10)	C18—C19—H19A	119.7
C6—C5—C4	119.01 (13)	C19—C20—C21	118.85 (11)
C6—C5—H5A	120.5	C19—C20—C23	120.83 (11)
C4—C5—H5A	120.5	C21—C20—C23	120.31 (12)
C5—C6—C7	121.04 (12)	C22—C21—C20	121.23 (12)
C5—C6—H6A	119.5	C22—C21—H21A	119.4
C7—C6—H6A	119.5	C20—C21—H21A	119.4
C8—C7—C6	121.29 (12)	C21—C22—C17	118.83 (11)
C8—C7—H7A	119.4	C21—C22—H22A	120.6
C6—C7—H7A	119.4	C17—C22—H22A	120.6
C7—C8—C9	117.26 (12)	C20—C23—H23A	109.5
C7—C8—H8A	121.4	C20—C23—H23B	109.5
C9—C8—H8A	121.4	H23A—C23—H23B	109.5
C8—C9—C4	122.62 (11)	C20—C23—H23C	109.5
C8—C9—N1	127.30 (11)	H23A—C23—H23C	109.5
C4—C9—N1	110.05 (10)	H23B—C23—H23C	109.5
			
O1—S1—N1—C10	42.28 (10)	C2—C3—C10—N2	0.36 (14)
O2—S1—N1—C10	171.37 (9)	C4—C3—C10—N2	−177.43 (10)
C11—S1—N1—C10	−72.74 (10)	C2—C3—C10—N1	175.95 (10)
O1—S1—N1—C9	174.84 (9)	C4—C3—C10—N1	−1.84 (13)
O2—S1—N1—C9	−56.07 (10)	C9—N1—C10—N2	176.61 (13)
C11—S1—N1—C9	59.82 (10)	S1—N1—C10—N2	−43.72 (18)
C10—N2—C1—C2	0.64 (13)	C9—N1—C10—C3	2.39 (13)
C10—N2—C1—S2	175.91 (9)	S1—N1—C10—C3	142.06 (9)
O4—S2—C1—C2	−20.80 (13)	O1—S1—C11—C16	154.12 (10)
O3—S2—C1—C2	−151.78 (11)	O2—S1—C11—C16	19.79 (12)
C17—S2—C1—C2	93.73 (12)	N1—S1—C11—C16	−94.18 (11)
O4—S2—C1—N2	164.83 (9)	O1—S1—C11—C12	−25.73 (11)
O3—S2—C1—N2	33.85 (11)	O2—S1—C11—C12	−160.07 (10)
C17—S2—C1—N2	−80.63 (10)	N1—S1—C11—C12	85.96 (11)
N2—C1—C2—C3	−0.43 (14)	C16—C11—C12—C13	−0.25 (19)
S2—C1—C2—C3	−175.30 (9)	S1—C11—C12—C13	179.61 (10)
C1—C2—C3—C10	0.05 (13)	C11—C12—C13—C14	−0.3 (2)
C1—C2—C3—C4	176.14 (18)	C12—C13—C14—C15	0.4 (2)
C10—C3—C4—C5	179.76 (14)	C13—C14—C15—C16	0.0 (2)
C2—C3—C4—C5	3.7 (3)	C14—C15—C16—C11	−0.49 (19)
C10—C3—C4—C9	0.47 (13)	C12—C11—C16—C15	0.64 (19)
C2—C3—C4—C9	−175.62 (18)	S1—C11—C16—C15	−179.22 (10)
C9—C4—C5—C6	−0.39 (18)	O4—S2—C17—C18	12.96 (11)
C3—C4—C5—C6	−179.62 (13)	O3—S2—C17—C18	143.93 (9)
C4—C5—C6—C7	0.5 (2)	C1—S2—C17—C18	−102.15 (10)
C5—C6—C7—C8	0.1 (2)	O4—S2—C17—C22	−168.94 (9)
C6—C7—C8—C9	−0.90 (19)	O3—S2—C17—C22	−37.97 (11)
C7—C8—C9—C4	1.05 (18)	C1—S2—C17—C22	75.95 (11)
C7—C8—C9—N1	178.71 (12)	C22—C17—C18—C19	1.07 (17)
C5—C4—C9—C8	−0.41 (18)	S2—C17—C18—C19	179.16 (9)
C3—C4—C9—C8	179.01 (11)	C17—C18—C19—C20	−1.00 (18)
C5—C4—C9—N1	−178.44 (11)	C18—C19—C20—C21	−0.28 (18)
C3—C4—C9—N1	0.99 (13)	C18—C19—C20—C23	179.65 (11)
C10—N1—C9—C8	−179.91 (11)	C19—C20—C21—C22	1.53 (18)
S1—N1—C9—C8	41.26 (16)	C23—C20—C21—C22	−178.40 (11)
C10—N1—C9—C4	−2.01 (12)	C20—C21—C22—C17	−1.47 (18)
S1—N1—C9—C4	−140.84 (9)	C18—C17—C22—C21	0.15 (17)
C1—N2—C10—C3	−0.61 (13)	S2—C17—C22—C21	−177.91 (9)
C1—N2—C10—N1	−174.88 (12)		

### Hydrogen-bond geometry (Å, °)

**Table:**

*D*—H···*A*	*D*—H	H···*A*	*D*···*A*	*D*—H···*A*
N2—H2B···O3^i^	0.88	2.06	2.9244 (14)	167
C13—H13A···O2^ii^	0.95	2.53	3.2125 (15)	129
C22—H22A···O3^i^	0.95	2.45	3.3786 (15)	165

Symmetry codes: (i) −*x*+1, −*y*, −*z*+1; (ii) *x*+1, *y*, *z*.
